# Editorial: Multilateral Interactions in the Rhizosphere

**DOI:** 10.3389/fmicb.2021.798728

**Published:** 2021-12-07

**Authors:** Soumitra Paul Chowdhury, László Kredics, Fred O. Asiegbu, Beatriz Lagunas, Adam Schikora

**Affiliations:** ^1^Institute for Network Biology, Helmholtz Zentrum München—German Research Center for Environmental Health (GmbH), Neuherberg, Germany; ^2^Department of Microbiology, Faculty of Science and Informatics, University of Szeged, Szeged, Hungary; ^3^Department of Forest Sciences, University of Helsinki, Helsinki, Finland; ^4^School of Life Sciences, University of Warwick, Coventry, United Kingdom; ^5^Julius Kühn Institute (JKI)—Federal Research Centre for Cultivated Plants, Institute for Epidemiology and Pathogen Diagnostics,Braunschweig, Germany

**Keywords:** rhizosphere, plant-microbe interaction, PGPR—plant growth-promoting rhizobacteria, induced systemic resistance, quorum sensing (QS)

Interactions between plants and diverse microorganisms colonizing their rhizosphere play a central role in determining nature of the relationship. The plant host fitness as well as the microorganisms are influenced by the outcome of such interactions. Environmental and ecological factors leading to perturbations or disruption of this balanced relationship have also a significant impact. The plant rhizosphere is a complex ecosystem serving as a niche for diverse microorganisms (bacteria, archaea, fungi), nematodes and other organisms. Within the rhizosphere the root exudates have a dual function, influencing nutrient availability and organisms in the vicinity of the root, on one hand. On the other, many microorganisms produce phytohormones that alter the root architecture or other compounds, which affect nutrient availability and thereby the competition between neighboring plants. Sometimes their presence can be beneficial for their host plant since they suppress the growth of phytopathogenic microorganisms. Some other rhizosphere microorganisms such as rhizobacteria and some fungi promote directly plant growth or stimulate the plant immune system. All these phenomena have potential practical applications in agriculture.

Even though we know a lot about the direct impact of individual microbial molecules on the plant itself, the exact mechanisms underpinning the action of complex microbial inoculants are not yet completely understood. It is however widely accepted that both below- and aboveground plant native microbiomes will have impact on the outcome of such interaction (Vishwakarma et al.). Nowadays, the microbial inocula used in order to prevent soil-borne diseases become rather complex and products based on single-strains are being replaced by biological consortia (Niu et al.). The effect on the plant immune system was very often in focus of the application of such complex microbial inoculants. Induced Systemic Resistance and priming for enhanced resistance have been practiced in agriculture for many years, and today the application of novel biotechnological advances is helping to provide better insights into the functional and ecological aspects. Nonetheless, how complex situations, e.g., multiple bacterial molecules influence the plant, remains unclear. It seems though, that complex bacterial quorum sensing molecules from the *N*-acyl homoserine lactones group induce priming for enhanced resistance (Shrestha et al.). An impact on the resistance to pathogens was observed also in the tripartite interaction between potato, arbuscular mycorrhiza fungi and potato virus Y (Deja-Sikora et al.). Interestingly, the presence of mycorrhiza reduced the production of free oxygen radicals, otherwise induced by the virus, even in PVY-infected plants. These results suggest that mycorrhizal fungi could mask the viral infection and promote asymptomatic growth. In addition to the impact of microbial partners on plant physiology, the effect of the plant's altered physiological response on the microbial community was also observable. This phenomenon was studied in drought-tolerant transgenic sugarcane, which can attract a different microbiome than its wild type parental line, illustrating also how the genotype of the host plant influences the microorganisms in its rhizosphere (Zhao et al.). Another example of a very specific and close association between plant and microorganisms, is the canonical symbiosis between legumes and rhizobia. These very specific associations depend on a selective rhizosphere communication between the bacterium and the legume plant. This communication includes multiple levels and both partners can influence the outcome. The constantly increasing knowledge of molecular mechanisms employed by both associates provides us with new opportunities to use and to understand this phenomenon (Walker et al.).

When discussing interactions in the rhizosphere, not only interactions between the plant and microorganisms seem important, also the structure and diversity of soil microbial community, which acts as the reservoir of microorganisms, are important because, beneficial or antagonistic interactions among microorganisms themselves are crucial. So does the edaphic factor. This was illustrated by the observation that in some cases the growing site and the agricultural practice are two major driving forces, which shape the rhizosphere community. Namely, in an experiment focused on *Brassica napus* only one bacterium was common among different experimental setups (Floc'h et al.). Several plant protection products have been developed using different *Bacillus* species. The performance however, largely depends on their interactions with competing rhizosphere microorganisms. How such plant-beneficial inocula react to other microorganisms is not always understood, and bridging this knowledge gap would improve the product development (Andrić et al.). A good example of how one strain may actually inhibit the function of another beneficial bacterium, is the quorum quenching activity of *Bacillus subtilis* on the N-fixing *Ensifer meliloti*, which diminishes its symbiotic activity (Rosier et al.). Particular bacterial community members, as well as the availability of nutrients, especially that of phosphorous, were also key factors in the tripartite association between plant, truffles and the soil microbiota (Zhang et al.), reflecting in addition the role of edaphic factors.

The complex systems assessed in the above-mentioned studies require a good experimental design and possibilities to control many different variables. This is possible in growth chamber studies under controlled conditions, the choice of an appropriate growing chamber seems therefore crucial and has been addressed in this issue in the study of Yee et al. Similarly, the colonization of the host plant by beneficial microorganisms necessitates quantification and documentation possibilities. Standardized frameworks assessing colonization efficacy and patterns could allow the comparison of different studies (Carroll et al.).

Much remains to be revealed about the multifunctionality of the belowground interplay among soil microorganisms and plant roots and the influence on the aboveground plant parts. Only through the understanding of these interactions will we be able to manage processes in this highly dynamic compartment to our benefit and enhance sustainable ecosystem functioning and crop production. Learning from natural ecosystems and employing targeted approaches which lead to enhanced plant productivity in agroecosystems will be of tremendous importance in the coming years and decades.

## Author Contributions

All authors listed have made a substantial, direct, and intellectual contribution to the work and approved it for publication.

## Conflict of Interest

The authors declare that the research was conducted in the absence of any commercial or financial relationships that could be construed as a potential conflict of interest.

## Publisher's Note

All claims expressed in this article are solely those of the authors and do not necessarily represent those of their affiliated organizations, or those of the publisher, the editors and the reviewers. Any product that may be evaluated in this article, or claim that may be made by its manufacturer, is not guaranteed or endorsed by the publisher.

